# The evolution of UDP-glycosyl/glucuronosyltransferase 1E (UGT1E) genes in bird lineages is linked to feeding habits but UGT2 genes is not

**DOI:** 10.1371/journal.pone.0205266

**Published:** 2018-10-31

**Authors:** Yusuke K. Kawai, Yoshinori Ikenaka, Mayumi Ishizuka, Akira Kubota

**Affiliations:** 1 Department of Veterinary Medicine, Obihiro University of Agriculture and Veterinary Medicine, Obihiro, Hokkaido, Japan; 2 Laboratory of Toxicology, School of Veterinary Medicine, Hokkaido University, Sapporo, Hokkaido, Japan; 3 Water Research Group, Unit for Environmental Science and Management, North-West University, Potchefstroom, North West province, South Africa; Laboratoire de Biologie du Développement de Villefranche-sur-Mer, FRANCE

## Abstract

UDP-glycosyltransferase (UGT) catalyzes the transfer of glycosyl groups (e.g., glucuronic acid) to exogenous or endogenous chemicals and plays an important role in conjugation reactions. In vertebrates, UGT genes are divided into 5 families: UGT1, UGT2, UGT3, UGT5, and UGT8. Among these UGT enzymes, UGT1 and UGT2 enzymes are known to be important xenobiotic metabolizing enzymes in mammals. However, little is known about UGT1 and UGT2 genes in avian species. In this study, we therefore aimed to classify avian UGT1 and UGT2 genes based on their evolutionary relationships. We also investigated the association between UGT molecular evolution and ecological factors, specifically feeding habits, habitat, and migration. By examining the genomes of 43 avian species with differing ecology, we showed that avian UGT1E genes are divided into 6 groups and UGT2 genes into 3 groups. Correlations between UGT gene count and ecological factors suggested that the number of UGT1E genes is decreasing in carnivorous species. Estimates of selection pressure also support the hypothesis that diet influenced avian UGT1E gene evolution, similar to mammalian UGT1A and UGT2B genes.

## Introduction

UDP-glycosyltransferase (UGT) catalyzes the transfer of glycosyl groups (including glucuronic acid, glucose, glycoside, and galactose) to exogenous or endogenous chemicals [1]. Vertebrate UGT genes are classified into 5 groups: 1, 2, 3, 5, and 8 [2–4]. In each UGT subfamily, genes were amplified by tandem duplication, with some of them specifically amplified (or even absent) of in lineages. For instance, UGT5 family genes are only in teleost fishes [3], while the UGT3 family is absent in chicken, turkey, and zebra finch [4], suggesting that avian species have 3 UGT families (UGT1, UGT2, and UGT8). Among these UGT enzymes, UGT1, UGT2, and UGT5 family enzymes were reported to catalyze the exogenous chemicals in zebrafish [5]. UGT1 and UGT2 family enzymes are known to be related to xenobiotic metabolism in mammals as well[6]. Therefore, UGT1 and UGT2 enzyes would be considered to be related to xenobiotic metabolism also in avian species.

UGT1 enzymes use UDP-glucronic acid (UDPGA) to engage in glucronic-acid transfer [1, 6]. Functional differences in UGT1As derive from a variable first exon among the 5 exons that constitute these genes (exon2-5 are conserved), with a prime example being immunoglobulin variation generating a robust immune defense[7]. In humans, UGT1A genes are divided into two functional groups, although this division is imperfect because of the genes’ complex roles. Bilirubin-like-associated enzymes (UGT1A1, UGT1A3, UGT1A4, UGT1A5) comprise the first group, whereas phenol-like-associated enzymes (UGT1A6, UGT1A7, UGT1A8, UGT1A9, UGT1A10) comprise the second [6]. Although nearly all mammals possess UGT1A1 for conjugating bilirubin, mammalian UGT1A6 (important for xenobiotic metabolism) has become a pseudogene in carnivorous mammals, including cats, brown hyenas, and northern fur seals [8, 9], likely because their diet does not contain harmful plant compounds. Moreover, our previous study indicated that the number of UGT1A genes is decreasing in carnivorous mammals [9].

Similar to UGT1, UGT2 enzymes use UDP-glucronic acid (UDPGA) [1]. In humans, 6 exons encode UGT2A1, 2A2, and 2A3; exons 2-6 are shared in UGT2A1 and 2A2, whereas UGT2A3 has only unique exons [2]. Although UGT2A1 and UGT2A2 are highly active in bile acid glucuronidation [10], UGT2A genes are mainly expressed in the nasal epithelium [11, 12] and are also known to metabolize steroids [13]. Mammalian UGT2Bs are composed of 6 separately coded exons [2] and are abundantly expressed in the liver [1]. Human UGT2B enzymes conjugate endogenous compounds such as steroid hormones, retinoids, and fatty acids, as well as exogenous compounds including morphine, zidovudine, and nonsteroidal anti-inflammatory drugs [6]. Previously, we reported that a UGT2B31-like gene in Felidae has become a pseudogene and that similar to UGT1A, UGT2B genes have decreased in carnivorous mammals [14].

Several reports have examined the relationship of UGT1 and UGT2 genes among vertebrates [3, 4] to obtain a better understanding of molecular evolution. For instance, zebra finch UGT genes were evaluated to determine the evolutionary relationships of vertebrate UGT1 and UGT2 [3]. Similarly, the genomic structures of vertebrate UGT1 and UGT2 genes [4] were uncovered using data from chicken, turkey, zebra finch, and other vertebrate genomes. However there is no report on the classification of comprehensive avian UGT genes based on the evolutionary relationship to other vertebrate UGTs.

In this study, we performed phylogenetic and synteny analyses to classify avian UGT1 and UGT2 genes, using data from 43 avian species representing 32 orders. Moreover, we aimed to clarify UGT evolution in birds by investigating the influence of key ecological factors (feeding habit, habitat, and migration). Our analyses yielded the first comprehensive classification of UGT1 and UGT2 genes in birds and confirmed that feeding habit (specifically carnivory) influenced the evolution of this gene family.

## Materials and methods

### UGT gene sequences

To characterize evolutionary diversity in avian UGT genes, we performed TBLASTN searches [15] on 43 sequenced avian RNA sequences (S1 Table, S1 Fig). Each species’ genome was obtained from the March 2017 GenBank refseq database with e-value < 1e-2 as the identity threshold. Query sequences were 154 individual UGT protein sequences (S1 Data), annotated in Ensembl [16] (release 87) as UDP-glucuronosyltransferase, UDP-glycosyltransferase, or UDP-galactosetransferase for zebrafish (*Danio rerio*), western clawed frog (*Xenopus tropicalis*), green anole (*Anolis carolinensis*), mouse (*Mus musculus*) and human (*Homo sapiens*). We excluded genes with obviously different annotation from BLAST results. Sequences containing multiple genes in one annotation were divided into multiple genes (S2 Table). Names of UGT genes followed the guidelines from the UGT nomenclature committee (https://prime.vetmed.wsu.edu/resources/udp-glucuronsyltransferase-homepage).

### Synteny analysis

The chromosomal location of annotated genes was determined using genomic data from 43 bird species in Genbank (accession numbers in S2 Table). Human, mouse, green anole, western clawed frog, and zebrafish Ensembl gene locations were also used. Graphical representations of gene location were generated with the genoPlotR package [17] in R version 3.3.2 (R Core Team 2016).

### Phylogenetic analysis

Gene location, maximum likelihood (ML) phylogenetic analysis, and BLASTn searches were used for classifying UGT families and selecting UGT1 and UGT2 genes for Bayesian phylogenetic analysis. UGT1 and UGT2 genes were divided into exon1 and other exons, and then analyzed separately for phylogeny construction. Amino acid sequences were aligned in MAFFT version 7.2 [18] with the auto option and trimmed in trimAl [19] with the automated1 option. For model selection and phylogenetic analysis of UGT1 exon1, sequences with >200 bp and no gaps above 15 bp were chosen (see supplementary information: S1–S4 Files). The best-fit model was selected using the Bayes information criterion (BIC) calculated by CodeML on Aminosan [20, 21]. Phylogenetic analysis on each UGT family (UGT1 and UGT2) was performed in MrBayes5D [22–25] using 4 chains (3 heated, 1 cold). Models, MCMC generations, and burn-in generations are shown in S3 Table. Tracer 1.6 [26] was used to check for stabilization and convergence between runs.

### Phylogenetic generalized least square analysis

To determine whether the UGT gene count was correlated with ecology, the feeding habit, habitat, and migration status of each bird species was first classified based on Almeida et al. (S1 Table) [27]. To correct for autocorrelation, phylogenetic generalized least square (PGLS) regression was performed [28, 29] in R, with the gls function under a Brownian motion correlation structure (corBrownian). The ape [30], phytools [31], and geiger [32] packages were employed. The avian phylogenetic tree constructed by Prum et al. [33] was modified and used for PGLS analysis (S1 Fig). Model selection was performed with the Akaike information criterion (AIC).

### Estimating selection pressure

Feeding habits may have exerted differing levels of selective pressure on UGT1 genes. To examine these potential differences, the omega (nonsynonymous and synonymous; dN/dS) ratios of phylogenetic branches were estimated with CodeML in PAML4.9 [20], using codon alignment and tree topology from phylogenetic analysis (S2 Data). An omega ratio greater than, equal to, or less than one indicates positive, neutral, or negative selection pressure, respectively. The F3×4 codon frequency was applied for estimating omega and kapper (transition and transversion) ratios. Three models were applied for estimating omega ratio: homogenous the omega ratio in all feeding habits (carnivory, omnivory, and herbivory); different omega ratio between carnivorous species and other feeding habits; and different omega ratios across all feeding habits. Likelihood ratio tests were performed to determine model fit.

Omega ratios of UGT1 sites were also estimated, and those under positive selection were predicted using the Bayes-Empirical-Bayes (BEB) test implemented with CodeML in PAML4.9 [20].

## Results

### Classification of UGT families in bird lineages

The results of TBLASTN for 43 bird species using UGT genes from 5 other vertebrates (zebrafish, western clawed frog, green anole, mouse, and human) as queries, yielded 196 UGT1 and 108 UGT2 gene sequences (S2 Table). Nearly every tested bird species possessed UGT1 and UGT2 family genes.

### UGT1 family genes

**Fig 1:**
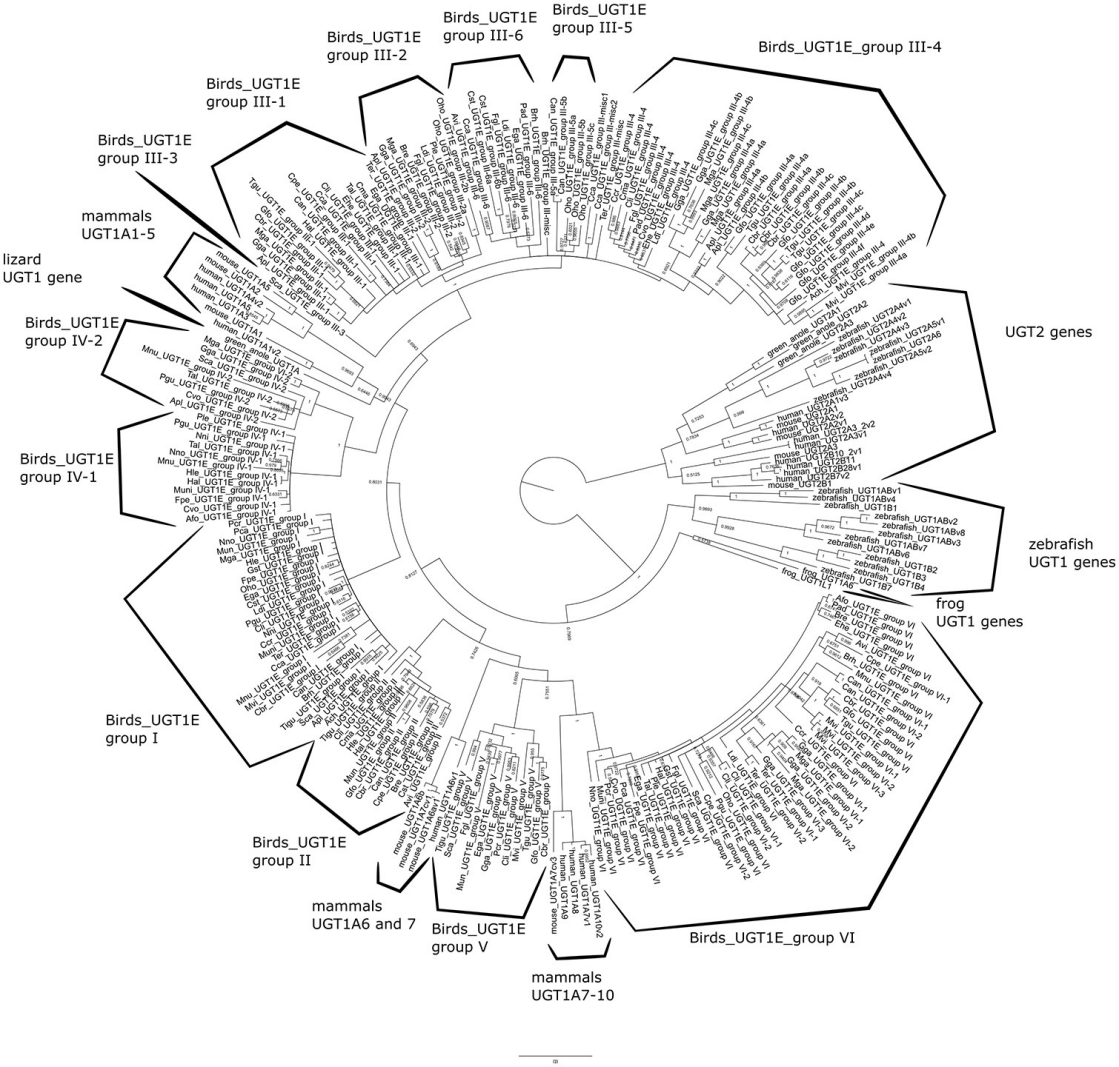
Phylogenetic classification of UGT1s in bird species. The phylogenetic tree of avian UGT1 exon1 was constructed in mrbayes5d, using sequences with >200 bp and without gaps >15 bp. Avian UGT1 exon1 were divided into 6 major groups. Groups III and IV were further divided into 6 and 2 subgroups, respectively.

**Fig 2:**
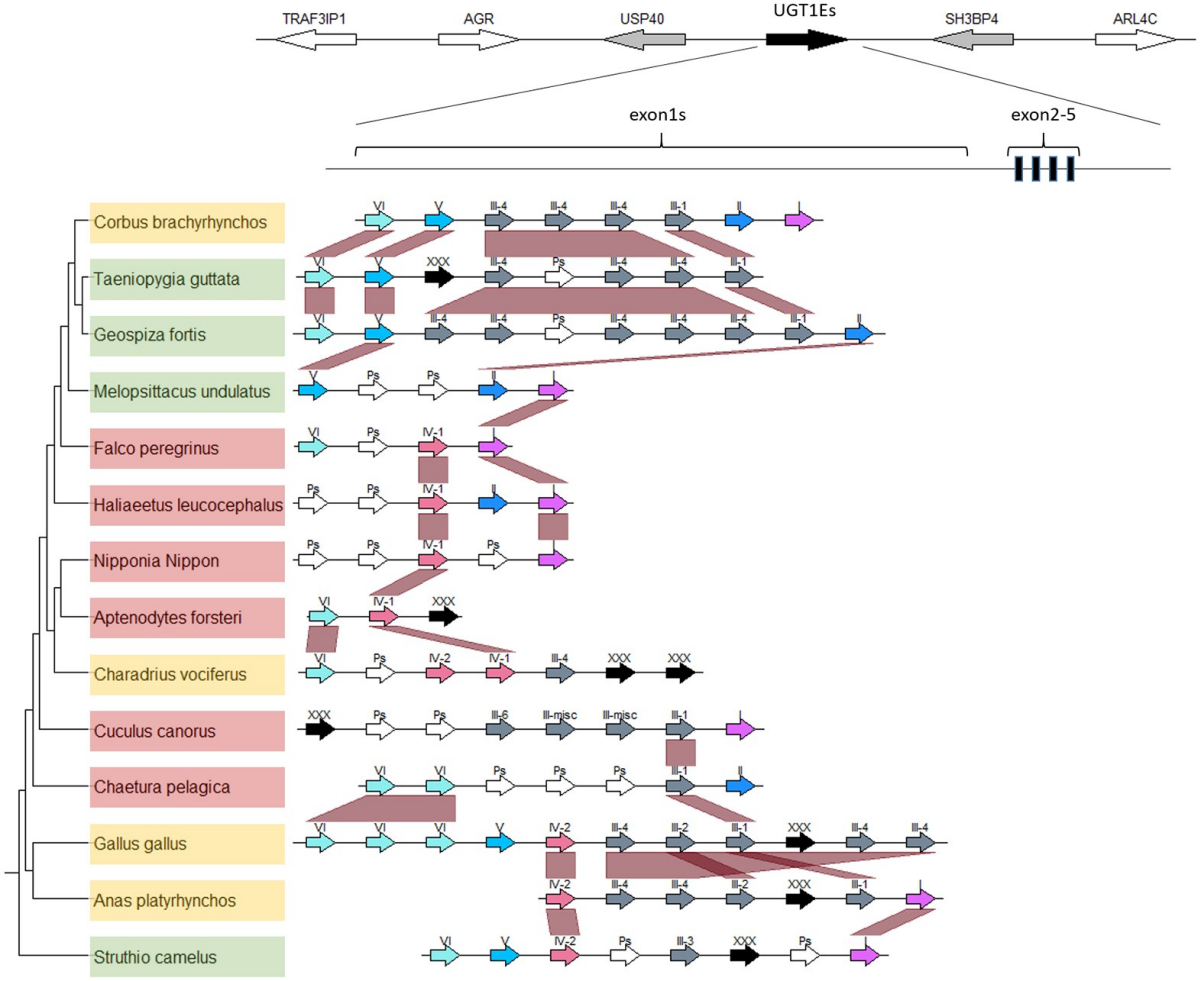
Synteny of UGT1s in bird species. Bird phylogeny and gene locations of UGT1 exon1 were visualized in genoplotR. Species are color-coded based on feeding habits: red, carnivorous; yellow, omnivorous; and green, herbivorous. UGT1 information was retrieved from a single contig. Avian UGT1Es were located between “USP40” and “SH3BP4”. Roman numbers on the arrows indicate the UGT1 group number, “Ps” indicates pseudogenes and “XXX” indicates unclassified (<200 bp) genes. Synteny of UGT1 exon1 was well conserved among bird species.

We chose “UGT1E” as the name for identified avian UGT1 genes, following guidelines from the UGT nomenclature committee (http://prime.vetmed.wsu.edu/resources/udp-glucuronsyltransferase-homepage). UGT1E family genes were divided into 6 groups with several subgroups, identified as “bird_UGT1E_group” based on gene location. Phylogenetic analysis revealed 6 clades based on the variable UGT1E exon1 regions (Fig 1), which were syntenic across birds (Fig 2). The remaining 4 exons (exon2-5) were shared across nearly all avian UGT1E genes (Fig 2); pigeons (*Columba livia*) were the only tested birds to possess duplicate exon2-5 (S2 Fig). Thus, in contrast to UGT1E exon1, UGT1E exons2-5 were generally conserved and reflected bird phylogeny. The phylogram of UGT1 exon2-5 indicated two clades: Palaeognathae and Neognathae. Among the Neognathae, crow (*Corvus brachyrhynchos*), zebra finch (*Taeniopygia guttata*), and Galapagos finch (*Geospiza fortis*) were divided from other species, but their posterior probability was <0.6, indicating that exons2-5 in Neognathae did not reflect avian phylogeny (S2 Fig).

### UGT2 family genes

**Fig 3:**
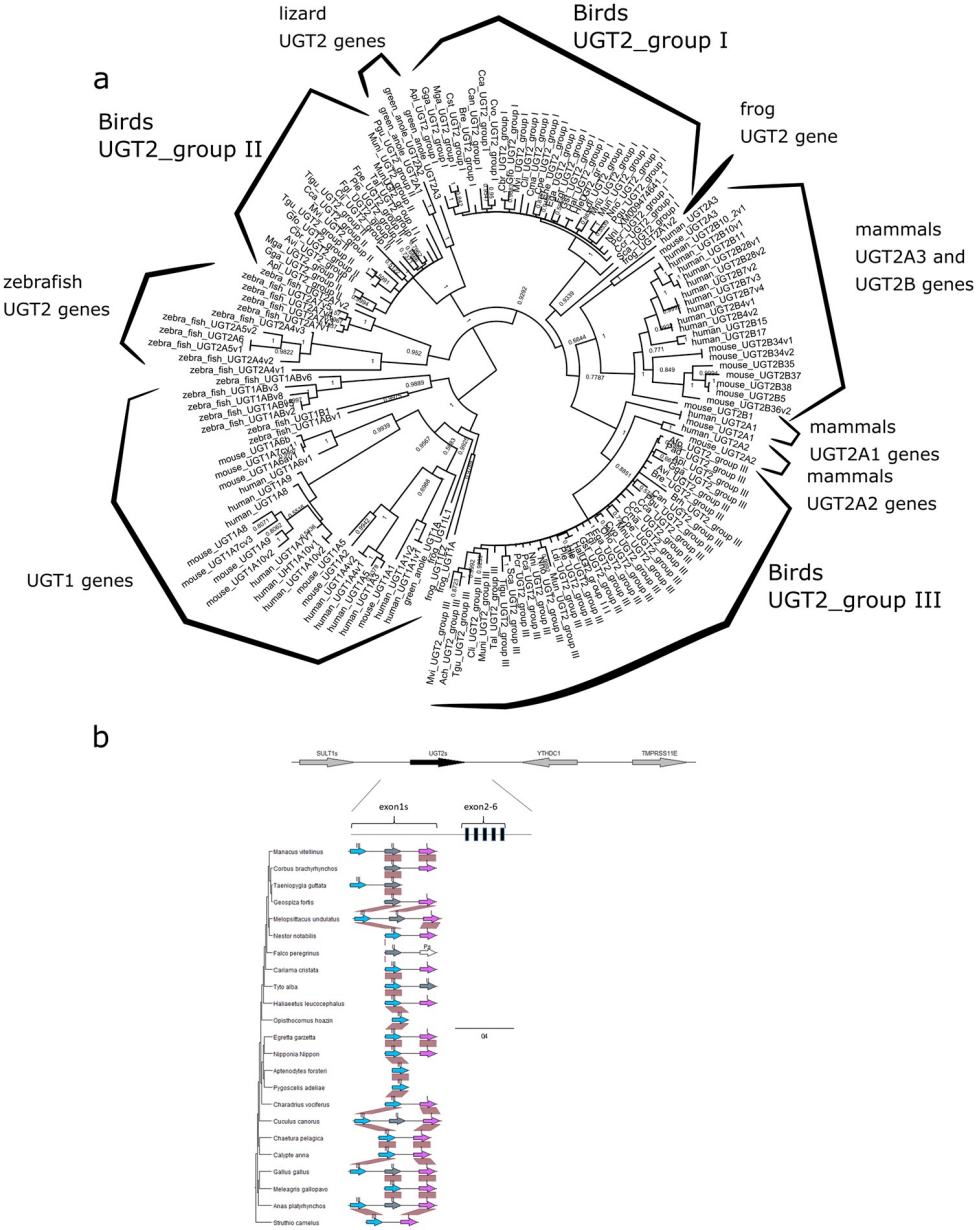
Classification of UGT2s in bird species. a) The phylogenetic tree based on avian UGT2 exon1 was constructed in mrbayes5d. Avian UGT2 exon1s are divided into 3 major groups (indicated with Roman numerals on arrows), with Group_I and Group_II forming a clade distinct from mammalian UGT2. However, bird UGT2 Group_III genes formed a single clade with mammalian UGT2A1 and UGT2A2. b) Avian phylogeny and gene locations of avian UGT2 genes were visualized in genoplotR. All UGT2 information was retrieved from a single contig. Avian UGT2s were located between “SULT1” and “YTHDC1.” Exons2-5 were shared across every avian species. Synteny of UGT2 exon1 was well conserved in birds.

Avian UGT2 genes contain 6 exons, with exons2-6 being shared (Fig 3b). In the phylogram of exons2-6, the mammalian UGT2A and UGT2B genes formed one clade, whereas avian UGT2 genes formed another (S3 Fig). In the latter clade, UGT2 exons2-6 did not correspond to avian phylogeny, including Palaeognathae and Neognathae. Avian UGT2 exon1 regions resulted in 3 clades (Fig 3a), termed “bird_UGT2_group.” Bird_UGT2_group_III genes formed one clade with mammalian UGT2A1 and UGT2A2 in the UGT2 exon1 phylogram. TBLASTN failed to detect UGT2 in *Tauraco erythrolophus* only. Two genes located around UGT2, “YTHDC1” and “SULT1,” were detected but had incomplete assembly.

### Relationships between the number of UGT genes and ecological factors of birds

**Fig 4:**
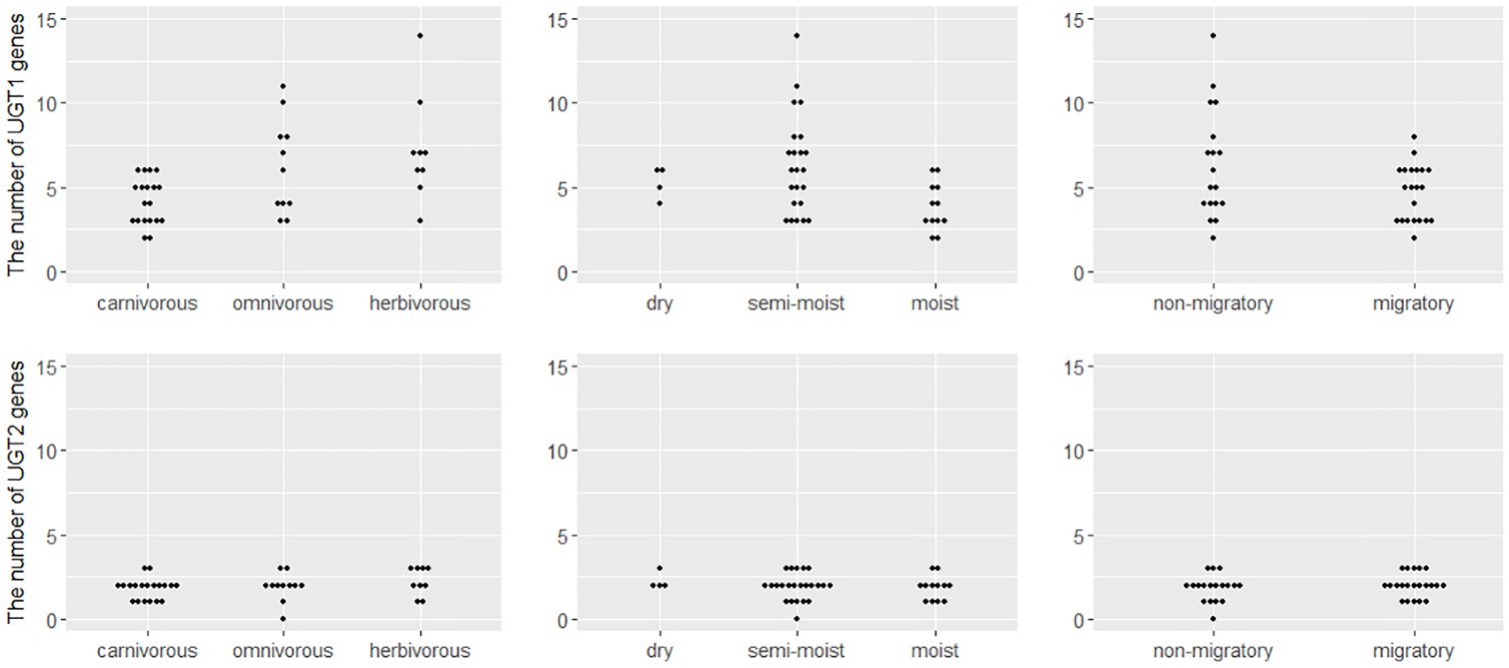
Relationship between the number of avian UGTs and ecological factors (feeding habit, habitat, and migration). The number of UGT1 genes differed across feeding habits (carnivory, herbivory, and omnivory).

Our analyses indicated that habitat and migration did not significantly impact the number of UGT genes, but feeding habits did (Fig 4). The best PGLS model (lowest AIC) indicated that carnivorous species had a lower UGT1E count than omnivorous and herbivorous species (Table 1). However, the number of UGT2 genes had no clear relationship to feeding habits.

**Table 1:** Phylogenetic generalized least square (PGLS) analysis.

Model	The number of parameters	Coefficients	AIC
Carivorous, Omnivorous = Herbivorous	2	4.311210, 6.487261	198.6751
Carivorous, Omnivorous, Herbivorous	3	4.390776, 6.150674, 7.182295	199.3981
Carivorous = Omnivorous, Herbivorous	2	5.443538, 7.229325	201.2292
Carivorous = Omnivorous = Herbivorous	1	5.798011	203.3232
Carivorous = Herbivorous, Omnivorous	2	5.586517, 6.022803	205.0414

The best-fit model (lowest Akaike Information Criterion, AIC) showed a significant effect of feeding habit, with carnivorous species possessing fewer avian UGT1 genes than omnivorous or herbivorous species.

### Estimating the selection pressure exerted by feeding habit on UGT1E exon1

The homogenous model estimated the omega ratio to be 0.392. In the model separating carnivory and other feeding habits, omega ratios were estimated as 0.329 (carnivores) and 0.436 (herbivores, omnivores). In the third model separating all three feeding habits, omega ratios were estimated as 0.329 (carnivores), 0.409 (omnivores), and 0.469 (herbivores). The likelihood ratio test indicated that omega ratios were significantly different across feeding habitats (*p* < 0.05).

### Detecting positive selection sites on UGT1E exon1

**Fig 5:**
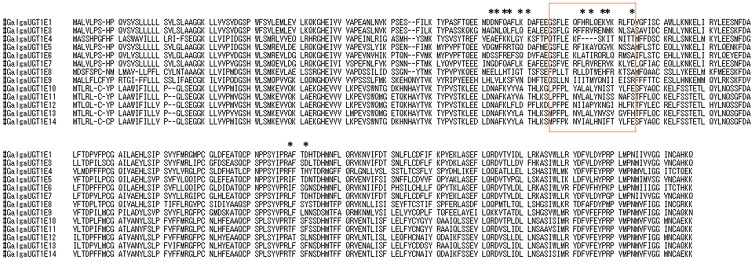
Positive selection on UGT1E exon1 sites in avian species. The sequences are chicken UGT1E genes. *: Positive selection sites estimated with Bayes Empirical Bayes (BEB) analysis using CodeML in PAML4.9; *p* < 0.05. Orange square indicates sites related to aglycone variation in human UGT1As [35].

The results of the BEB analysis [34] on estimated omega ratios (dN/dS) of UGT1A exon1 revealed 13 amino acid sites that were exposed to positive selection (Fig 5). Five of these 13 sites were located in a region related to aglycone variation in human UGT1As [35].

## Discussion

Our classification of avian UGT1 and UGT2 family genes clarified the evolutionary relationship of genes in bird lineages. First, UGT2 family genes were classified into 3 groups (Fig 3, S4 Fig). Mammalian UGT2B genes all exhibit variable exons1-6, whereas mammalian UGT2A1 and UGT2A2 genes share exons2-6 [2]. Simlarly to mammalian UGT2A1 and UGT2A2, avian UGT2 genes share exons2-6. Thus, depending on what exons are used to construct the phylogram, different interpretations of mammalian and avian relationships arise. Phylograms of UGT2 exons2-6 indicate that mammal and avian UGT2 genes are distinct (S3 Fig). This suggests that the ancestor of birds and mammals possessed one avian UGT2 exon2-6 set and it was duplicated in the mammalian lineage. In contrast, phylograms of UGT2 exon1 implied similarity between bird_UGT2_group_III and mammalian UGT2A1 and 2A2 genes (Fig 3). In humans, UGT2A1 and UGT2A2 are mainly expressed in the nasal epithelium [11, 12] in stable amounts. In contrast, UGT2B genes are abundantly expressed in the liver in variable amounts based on feeding habits [14]. Here, we observed relatively stable amounts of avian UGT2 genes, suggesting that in birds, UGT2 enzymes likely conjugate endogenous compounds and are more similar to mammalian UGT2A than UGT2B.

Our phylogram of UGT2 genes also appeared to be different from that of the previous study. A previous report indicated that mammalian UGT2A genes formed one clade, and each gene in humans has an orthologue in mice and other mammals [2, 12]. The reason why UGT2A3 genes did not form one clade in this study could involve differences in the exon region used.

**Fig 6:**
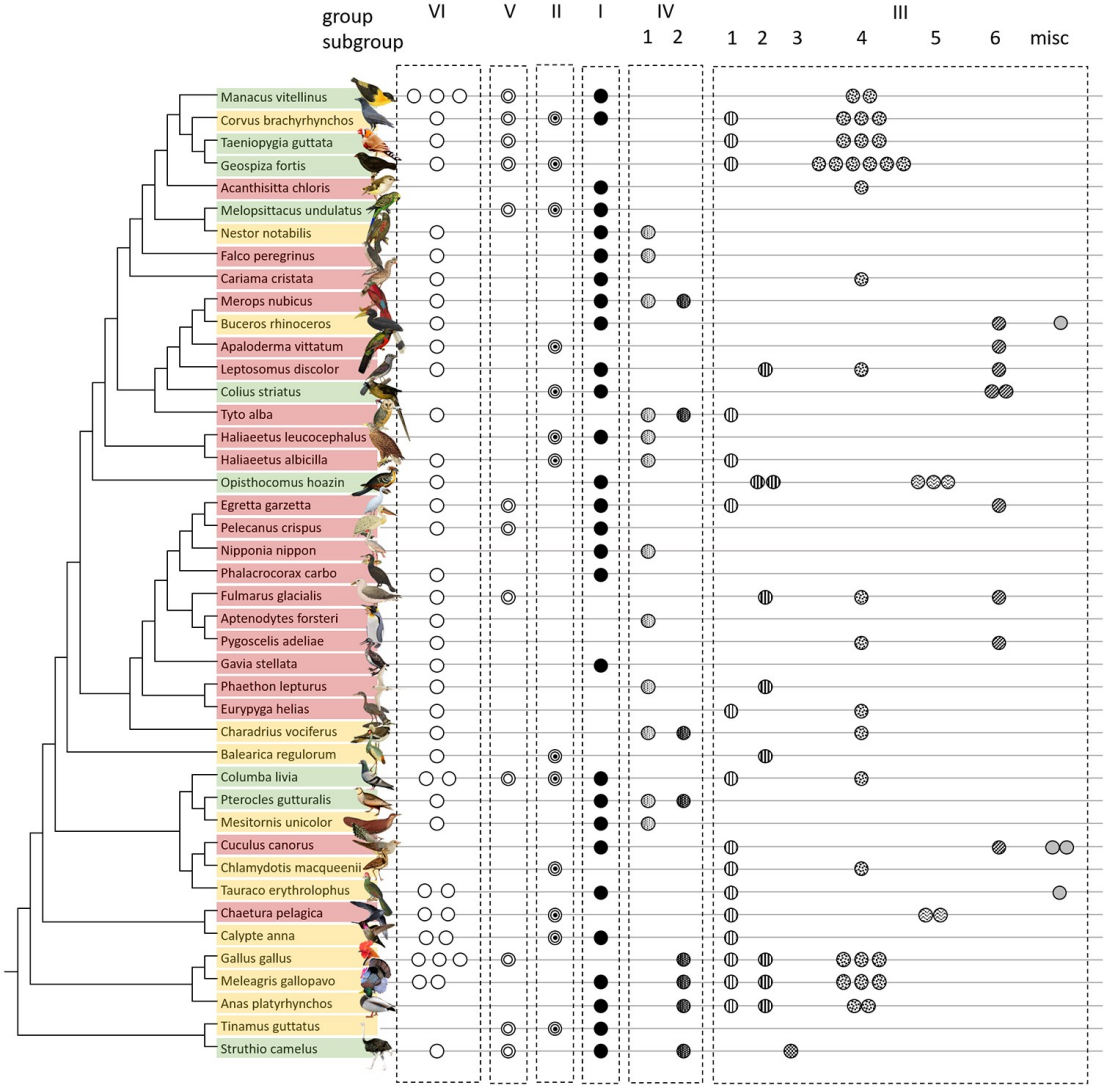
Classified UGT1E genes in bird species. Avian phylogenetic tree classifying UGT1 family genes (excluding those with sequences <200 bp). Species are color-coded based on feeding habits: red, carnivorous; yellow, omnivorous; and green, herbivorous. Illustrations of *Anas platyrhynchos*, *Aptenodytes forsteri*, *Columba livia*, *Gallus gallus*, *Meleagris gallopavo*, *Struthio camelus*, and *Taeniopygia guttata* were modified from the Togo picture gallery (http://togotv.dbcls.jp/ja/pics.html), licensed under CC-BY 4.0 Togo picture gallery by the Database Center for Life Science (DBCLS), Japan. Other illustrations were modified from public domain resources.

Some controversy exists in terms of nomenclature for avian UGT1 genes. Zebra finch UGT1 genes were named UGT1As based on evolutionary relationships [3]. However, the UGT nomenclature committee named UGT1 genes in chicken as UGT1Es based on their sequence similarity (https://prime.vetmed.wsu.edu/resources/udp-glucuronsyltransferase-homepage/current-nomenclature). In this study, we followed the second nomenclature guidelines and named UGT1 genes as UGT1Es. Our phylogenetic and synteny analyses classified UGT1 family genes into 6 major groups (Figs 1, 2 and 6). The results suggest that the avian common ancestor would have possessed 6 UGT1 genes that were subsequently duplicated in each lineage. Genomic organization also showed that some UGT1Es became pseudogenes in each lineage (Fig 2). This suggests that UGT1E genes underwent frequent duplication and loss in birth-and-death evolution. Notably, the number of UGT1E_group_III varies among birds and may be important for conjugating different exogenous compounds.

Our analysis found a significant relationship between UGT1E gene count and avian feeding habits, with carnivores possessing fewer UGT1Es than herbivores or omnivores Figs 6 and 4 and Table 1). When we examined the selection pressure on UGT1 exon1 to determine whether such differences were due to natural selection, we did not find evidence of positive selection on any phylogenetic branches (omega ratio > 1). However, the higher omega ratio in herbivorous species suggests that UGT1E enzymes play an important role in metabolizing toxic chemicals synthesized by plants.

However, few data on UGT1 structure are available to corroborate this potential function, even in humans. Available reports found a variable region around residues 105-131 in human UGT1As that appears to confer aglycone specificity [35] (Fig 5). This region is similar to the positively selected sites in avian UGT1Es, suggesting a link to aglycone specificity.

Indeed, some reports have described a similar relationship between xenobiotic metabolizing enzymes and feeding habits. Consistent with our study, carnivorous mammals, for example, also have fewer UGT1A genes [9]. However, we did not observe a correlation between UGT2 gene count and avian feeding habit, in contrast with mammalian UGT2 genes. Among mammals, the number of UGT2B genes in carnivorous species was lower than that in herbivorous and omnivorous species[14]. This difference suggests that UGT2 gene evolution in birds and mammals experienced different selective pressures.

Here, we did not find any link between other ecological factors (habitat, migration) and UGT gene count. Our findings on the influence of ecology thus partially contradict existing avian studies on the relationship between ecological factors and genes related to xenobiotic metabolism. For example, carnivorous birds or those living in wet habitats tend to possess fewer sensitive aryl hydrocarbon receptors (AhR), which regulate the expression of genes encoding xenobiotic metabolizing enzymes (e.g., UGT1s and CYP1s). Avian AhRs are divided into three genetic types: highly sensitive (Ile-324 and Ser-380), moderately sensitive (Ile-324 and Ala-380), and less sensitive to dioxin (Val-324 and Ala-380) [36, 37]. Certain species may have more of the third genetic type because they receive elevated levels of naturally occurring dioxins through the food web in specific habitats or under specific diets [38]. The lack of any observed relationship between UGTs and habitat in this study could be because UGTs are not directly involved in dealing with toxicity from dioxin-like compounds [36].

Previous research has also found that the omega ratio of avian cytochrome P450 (CYP), CYP2C23 (classically called CYP2H) and CYP2J_2, differs across feeding habits [27]; these enzymes are important for functionalization reactions in xenobiotic metabolism reactions. Among passerines, insectivores and granivores exhibit differing levels of ethoxyresorufin-O-deethylase (EROD) activity, which itself reflects CYP1A activity. Specifically, EROD activity is higher in insectivores than in granivores, possibly because some insects use defensive compounds from plants [39]. Still other reports have suggested that conjugation enzymes are important in nectar-eating birds for metabolizing nicotine, a process that varies in mechanism across avian species [40]. Together, these studies indicate that increased granularity in feeding classifications may yield a clearer picture regarding how diet influences UGT evolution. However, given the limited whole-genome data available at this point, we were unable to discriminate between insectivores and other carnivores, granivores and other herbivores, or nectarivores and other omnivores. Therefore, we recommend a focus on increasing whole-genome data for avian species to enhance investigations on the evolution of UGTs and other major genes in birds.

In this study, we confirmed ecological factors did not cause significant differences in the number of avian UGT2 genes. Given that mammalian UGT2 genes respond to ecological variation, our results suggest that different selective pressures influenced UGT2 evolution in birds versus mammals. However, UGT1 gene counts varied between feeding habits, with carnivores possessing significantly less than either herbivores or omnivores. Therefore, we conclude that diet exerted a clear effect on the evolution of avian UGT1E genes.

## Supporting information

S1 Fig Avian phylogenetic tree used in this study. We modified an existing phylogenetic tree constructed by Prum et al. [33].
(PNG)

S2 Fig Phylogeny of UGT1 exon2-5 regions. Avian UGT1 exon2-5 formed one clade distinct from other vertebrates. Palaeognathae and Neognathae formed two clades within the avian branch.
(PNG)

S3 Fig Phylogeny of UGT2 exon2-6 region. Avian UGT2 exons2-6 formed one clade distinct from the clade comprising mammalian UGT2A and UGT2B. Avian phylogenetic relationships (including Palaeognathae and Neognathae) were not reflected in the avian clade, however.
(TIF)

S4 Fig Classified UGT2 genes in bird species. A phylogenetic tree depicting how UGT2 family genes are classified.
(PNG)

S1 File Alignment of UGT1 exon1 for phylogenetic analysis. (TXT)

S2 File Alignment of UGT1 exons2-5 for phylogenetic analysis. (TXT)

S3 File Alignment of UGT2 exon1 for phylogenetic analysis. (TXT)

S4 File Alignment of UGT2 exons2-5 for phylogenetic analysis. (TXT)

S1 Data Query sequences for TBLASTN. (TXT)

S2 Data Codon alignment and tree topology for estimating omega ratio. (ZIP)

S1 Table Species information. Common name, scientific name, code used in this study, and ecological factors (feeding habit, habitat, and migration; based on Almeida et al. [27]).
(CSV)

S2 Table Accession numbers of avian UGT genes. Sequence data of avian UGT genes were obtained from GenBank.
(CSV)

S3 Table Models, Markov chain Monte Carlo (MCMC) generations, and burn-in generations in Bayesian phylogenetic analysis. (CSV)

## References

[1] Rowland A, Miners JO, Mackenzie PI. The UDP-glucuronosyltransferases: Their role in drug metabolism and detoxification. Int J Biochem Cell Biol. 2013;45(6):1121–1132. 10.1016/j.biocel.2013.02.019 23500526

[2] Mackenzie PI, Bock KW, Burchell B, Guillemette C, Ikushiro Si, Iyanagi T, et al Nomenclature update for the mammalian UDP glycosyltransferase (UGT) gene superfamily. Pharmacogenet Genomics 2005;15(10):677–85. 10.1097/01.fpc.0000173483.13689.56 16141793

[3] Huang H, Wu Q. Cloning and comparative analyses of the zebrafish Ugt repertoire reveal its evolutionary diversity. PloS One 2010;5(2):e9144 10.1371/journal.pone.0009144 20161780 PMC2819257

[4] Meech R, Miners JO, Lewis BC, Mackenzie PI. The glycosidation of xenobiotics and endogenous compounds: Versatility and redundancy in the UDP glycosyltransferase superfamily. Pharmacol Ther. 2012;134(2):200–18. 10.1016/j.pharmthera.2012.01.009 22322248

[5] Wang Y, Huang H, Wu Q. Characterization of the Zebrafish Ugt Repertoire Reveals a New Class of Drug-Metabolizing UDP Glucuronosyltransferases. Mol Pharmacol. 2014;86:62–75. 10.1124/mol.113.091462 24728488

[6] Guillemette C, Lévesque E, Harvey M, Bellemare J, Menard V. UGT genomic diversity: beyond gene duplication. Drug Metab Rev. 2010;42(1):24–44. 10.3109/03602530903210682 19857043

[7] Li C, Wu Q. Adaptive evolution of multiple-variable exons and structural diversity of drug-metabolizing enzymes. BMC Evol Biol. 2007;7:69 10.1186/1471-2148-7-69 17475008 PMC1885805

[8] Shrestha B, Reed JM, Starks PT, Kaufman GE, Goldstone JV, Roelke ME, et al Evolution of a major drug metabolizing enzyme defect in the domestic cat and other Felidae: phylogenetic timing and the role of hypercarnivory. PloS One 2011;6(3):e18046 10.1371/journal.pone.0018046 21464924 PMC3065456

[9] Kakehi M, Ikenaka Y, Nakayama SMM, Kawai YK, Watanabe KP, Mizukawa H, et al Uridine Diphosphate-Glucuronosyltransferase (UGT) xenobiotic metabolizing activity and genetic evolution in pinniped species. Toxicol Sci. 2015;147(2):360–369. 10.1093/toxsci/kfv144 26179383

[10] Perreault M, Gauthier-Landry L, Trottier J, Verreault M, Caron P, Finel M, et al The human UDP-glucuronosyltransferase UGT2A1 and UGT2A2 enzymes are highly active in bile acid glucuronidation. Drug Metab Dispos. 2013;41(9):1616–1620. 10.1124/dmd.113.052613 23756265 PMC3815633

[11] Jedlitschky G, Cassidy AJ, Sales M, Pratt N, Burchell B. Cloning and characterization of a novel human olfactory UDP-glucuronosyltransferase. Biochem J. 1999;340:837–843. 10.1042/0264-6021:3400837 10359671 PMC1220318

[12] Court MH, Hazarika S, Krishnaswamy S, Finel M, Williams JA. Novel polymorphic human UDP-glucuronosyltransferase 2A3: cloning, functional characterization of enzyme variants, comparative tissue expression, and gene induction. Mol Pharmacol. 2008;74(3):744–754. 10.1124/mol.108.045500 18523138 PMC2574548

[13] Chouinard S, Yueh MF, Tukey RH, Giton F, Fiet J, Pelletier G, Barbier O, Belanger A. Inactivation by UDP-glucuronosyltransferase enzymes: The end of androgen signaling. J Steroid Biochem Mol Biol. 2008;109:247–253. 10.1016/j.jsbmb.2008.03.016 18467088

[14] Kondo T, Ikenaka Y, Nakayama SMM, Kawai YK, Mizukawa H, Mitani Y, et al Uridine diphosphate-glucuronosyltransferase (UGT) 2B subfamily interspecies differences in carnivores. Toxicol Sci. 2017;. 10.1093/toxsci/kfx072 28453659

[15] Camacho C, Coulouris G, Avagyan V, Ma N, Papadopoulos J, Bealer K, et al BLAST plus: architecture and applications. BMC Bioinf. 2009;10(421):1 10.1186/1471-2105-10-421 PMC2803857 20003500

[16] Flicek P, Amode MR, Barrell D, Beal K, Billis K, Brent S, et al Ensembl 2014. Nucleic Acids Res. 2014;42(D1):749–755. 10.1093/nar/gkt1196 PMC3964975 24316576

[17] Guy L, Kultima JR, Andersson SGE, Quackenbush J. GenoPlotR: comparative gene and genome visualization in R. Bioinformatics 2011;27(13):2334–2335. 10.1093/bioinformatics/btq413 PMC2935412 20624783

[18] Katoh K, Standley DM. MAFFT Multiple Sequence Alignment Software Version 7: improvements in performance and usability article fast track. Mol Biol Evol. 2013;30(4):772–780. 10.1093/molbev/mst010 23329690 PMC3603318

[19] Capella-Gutiérrez S, Silla-Martínez JM, Gabaldón T. trimAl: A tool for automated alignment trimming in large-scale phylogenetic analyses. Bioinformatics 2009;25(15):1972–1973. 10.1093/bioinformatics/btp348 19505945 PMC2712344

[20] Yang Z. PAML 4: phylogenetic analysis by maximum likelihood. Mol Biol Evol. 2007;24(8):1586–91. 10.1093/molbev/msm088 17483113

[21] Tanabe AS. Kakusan4 and Aminosan: Two programs for comparing nonpartitioned, proportional and separate models for combined molecular phylogenetic analyses of multilocus sequence data. Mol Ecol Resour. 2011;11(5):914–921. 10.1111/j.1755-0998.2011.03021.x 21592310

[22] Huelsenbeck JP, Ronquist F. MRBAYES: Bayesian inference of phylogenetic trees. Bioinformatics 2001;17(8):754–755. 10.1093/bioinformatics/17.8.754 11524383

[23] Ronquist F, Huelsenbeck JP. MrBayes 3: Bayesian phylogenetic inference under mixed models. Bioinformatics 2003;19(12):1572–1574. 10.1093/bioinformatics/btg180 12912839

[24] Altekar G, Dwarkadas S, Huelsenbeck JP, Ronquist F. Parallel Metropolis coupled Markov chain Monte Carlo for Bayesian phylogenetic inference. Bioinformatics 2004;20(3):407–415. 10.1093/bioinformatics/btg427 14960467

[25] Tanabe AS. MrBayes5D. [cited 13 April 2017]. Available from: http://fifthdimension.jp/products/mrbayes5d.

[26] Rambaut A, Suchard MA, Xie D, Drummond AJ. Tracer v1.6. Available from: http://beast.bio.ed.ac.uk/Tracer.

[27] Almeida D, Maldonado E, Khan I, Silva L, Gilbert MTP, Zhang G, et al Whole genome identification, phylogeny and evolution of the cytochrome P450 family 2 (CYP2) sub-families in birds. Genome Biol Evol. 2016;2(4):evw041 10.1093/gbe/evw041 PMC4860681 26979796

[28] Rohlf JF. Comparative methods for the analysis of continuous variables: geometric interpretations. Evolution 2001;55(11):2143–2160. 10.1111/j.0014-3820.2001.tb00731.x 11794776

[29] Martins EP, Hansen TF. Phylogenies and the comparative method: a general approach to incorporating phylogenetic information into the analysis of interspecific data. Am Nat. 1997;149(4):646–667. 10.1086/286013

[30] Paradis E, Claude J, Strimmer K. APE: Analyses of Phylogenetics and Evolution in R language. Bioinformatics 2004;20(2):289–290. 10.1093/bioinformatics/btg412 14734327

[31] Revell LJ. phytools: an R package for phylogenetic comparative biology (and other things). Methods Ecol Evol. 2012;3:217–223. 10.1111/j.2041-210X.2011.00169.x

[32] Harmon LJ, Weir JT, Brock CD, Glor RE, Challenger W, Science A. GEIGER: investigating evolutionary radiations. Bioinformatics 2008;24(1):129–131. 10.1093/bioinformatics/btm538 18006550

[33] Prum RO, Berv JS, Dornburg A, Field DJ, Townsend JP, Lemmon EM, et al A comprehensive phylogeny of birds (Aves) using targeted next-generation DNA sequencing. Nature 2015;. 10.1038/nature15697 26444237

[34] Yang Z, Wong WSW, Nielsen R. Bayes empirical Bayes inference of amino acid sites under positive selection. Mol Biol Evol. 2005;22(4):1107–1118. 10.1093/molbev/msi097 15689528

[35] Locuson CW, Tracy TS. Comparative modelling of the human UDP-glucuronosyltransferases: insights into structure and mechanism. Xenobiotica 2007;37(2):155–168. 10.1080/00498250601129109 17484518

[36] Karchner SI, Franks DG, Kennedy SW, Hahn ME. The molecular basis for differential dioxin sensitivity in birds: Role of the aryl hydrocarbon receptor. Proc Nat Acad Sci USA. 2006;103:6252–6257. 10.1073/pnas.0509950103 16606854 PMC1435364

[37] Fujisawa N, Kawai YK, Nakayama SMM, Ikenaka Y, Yamamoto H, Ishizuka M. Dioxin sensitivity-related two critical amino acids of arylhydrocarbon receptor may not correlate with the taxonomy or phylogeny in avian species. J Vet Med Sci. 2013;75(12):1577–1583. 10.1292/jvms.13-0179 23912877 PMC3942959

[38] Hwang Jh, Park Jy, Park Hj, Bak Sm, Hirano M, Iwata H, et al Ecological factors drive natural selection pressure of avian aryl hydrocarbon receptor 1 genotypes. Sci Rep. 2016;6:27526 10.1038/srep27526 27283192 PMC4901312

[39] Rainio MJ, Kanerva M, Wahlberg N, Nikinmaa M, Eeva T. Variation of basal EROD activities in ten passerine bird species—relationships with diet and migration status. PLoS One. 2012;7(3):e33926 10.1371/journal.pone.0033926 22479477 PMC3315499

[40] Lerch-Henning S, Du Rand EE, Nicolson SW. Detoxification and elimination of nicotine by nectar-feeding birds. J Comp Physiol B. 2017;187(4):591–602. 10.1007/s00360-016-1055-4 28150179

