# Supplementary figures and images for: The evolution of UDP-glycosyl/glucuronosyltransferase 1E (UGT1E) genes in bird lineages is linked to feeding habits but UGT2 genes is not
Source: PLoS One. 2018 Oct 31;13(10):e0205266. doi: 10.1371/journal.pone.0205266 (PMC6209164; doi:10.1371/journal.pone.0205266)

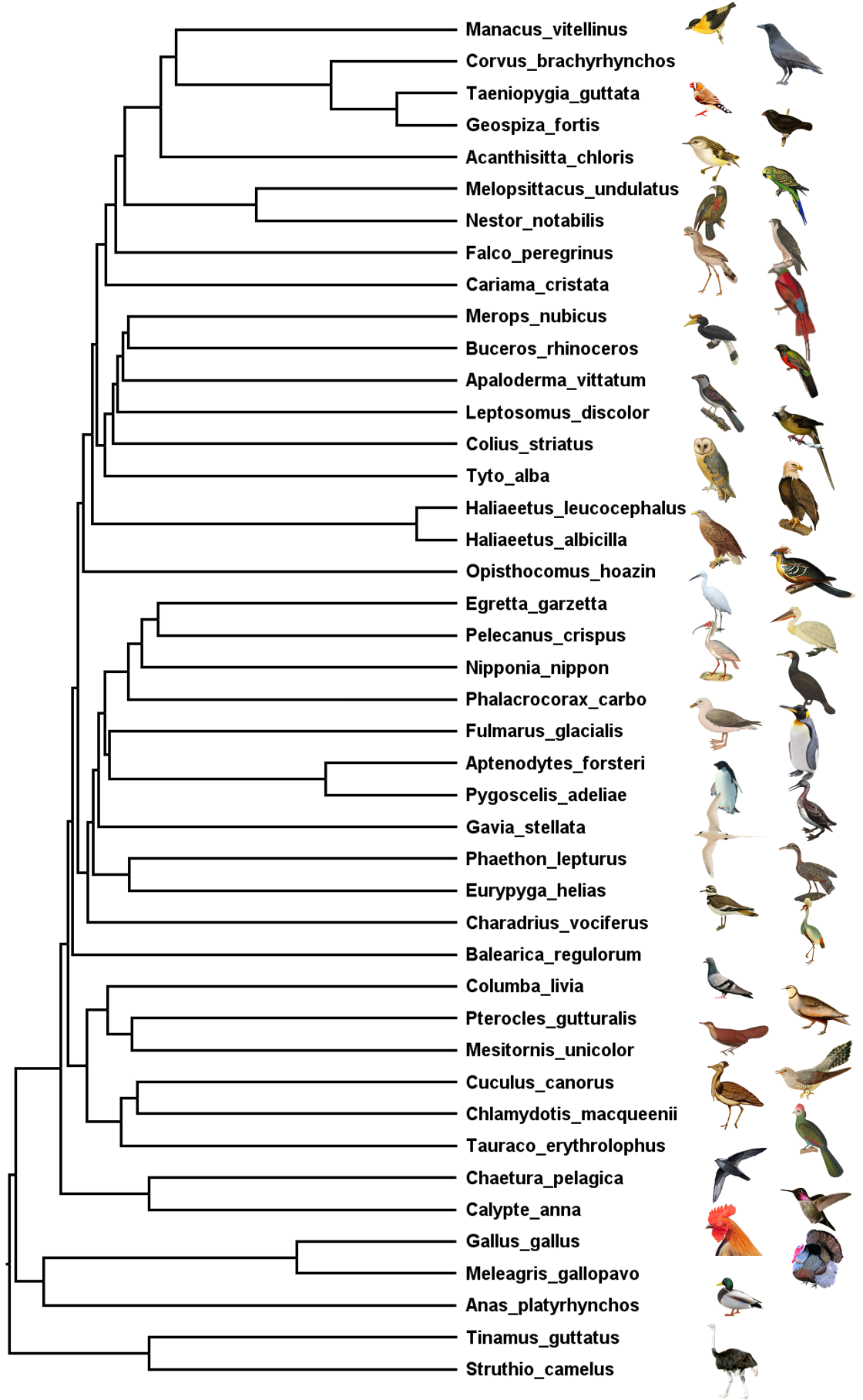

Supplement: S1 Fig — We modified an existing phylogenetic tree constructed by Prum et al. [33]. (PNG) [file pone.0205266.s001.png]

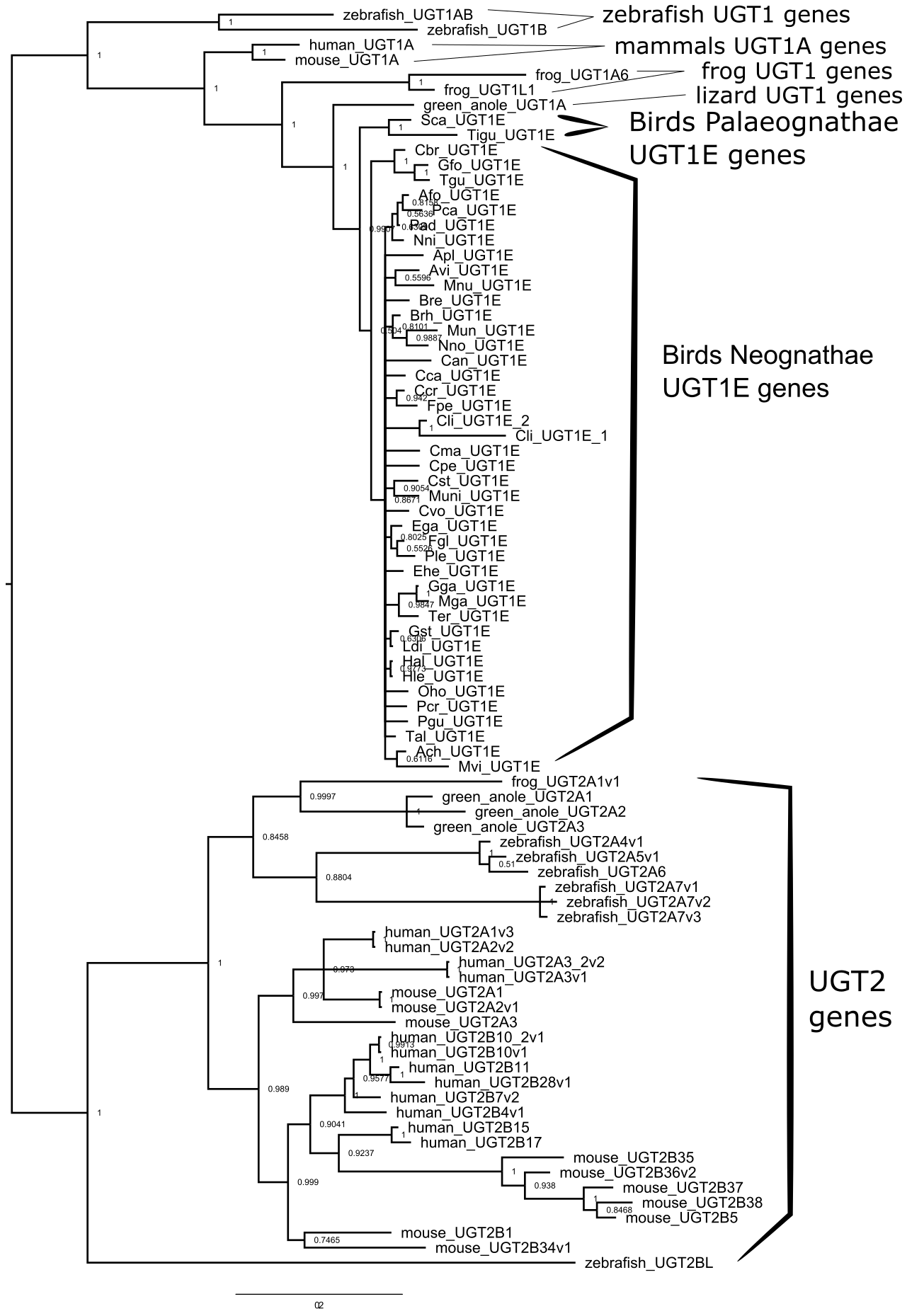

Supplement: S2 Fig — Avian UGT1 exon2-5 formed one clade distinct from other vertebrates. Palaeognathae and Neognathae formed two clades within the avian branch. (PNG) [file pone.0205266.s002.png]

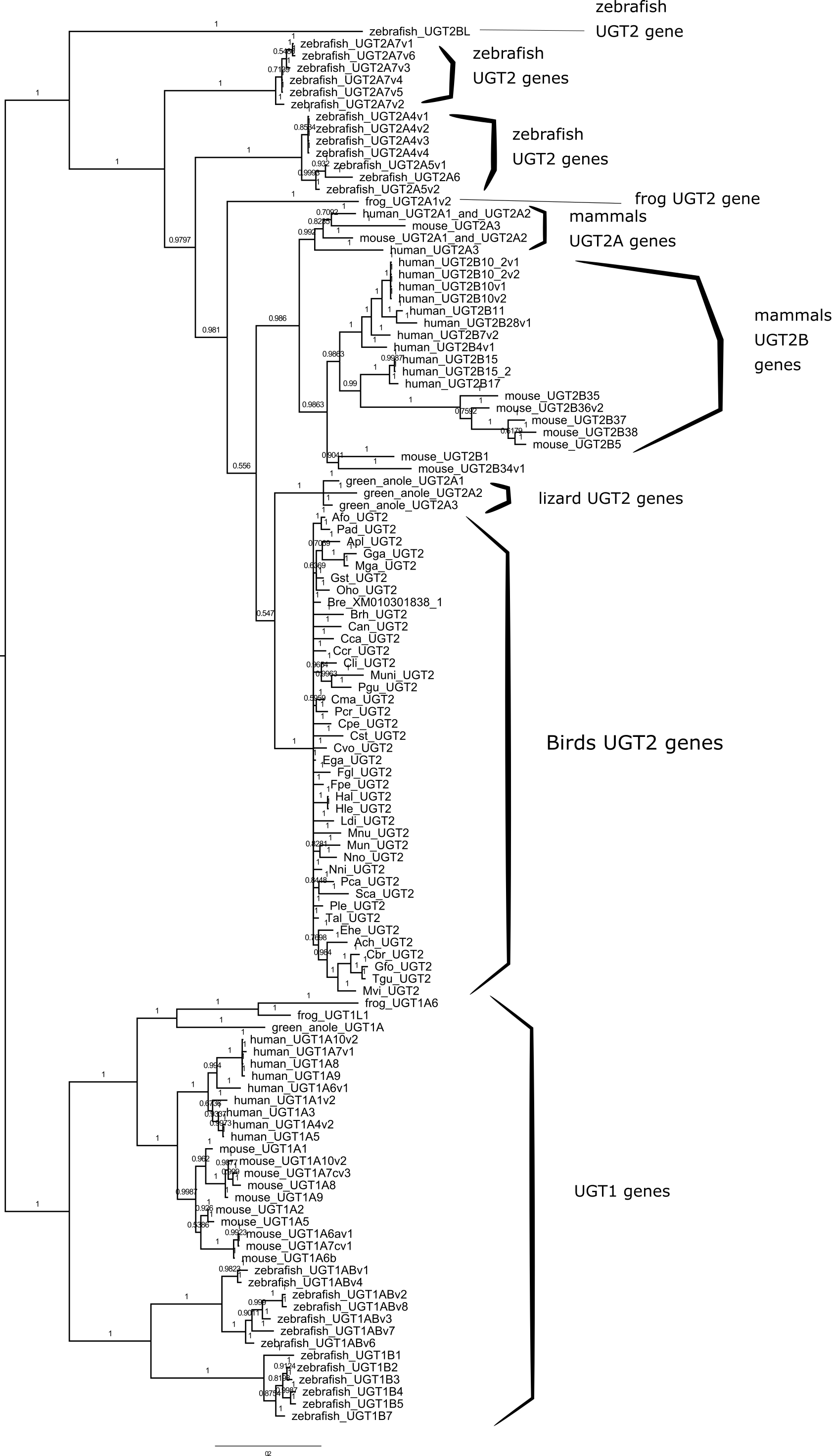

Supplement: S3 Fig — Avian UGT2 exons2-6 formed one clade distinct from the clade comprising mammalian UGT2A and UGT2B. Avian phylogenetic relationships (including Palaeognathae and Neognathae) were not reflected in the avian clade, however. (TIF) [file pone.0205266.s003.tif]

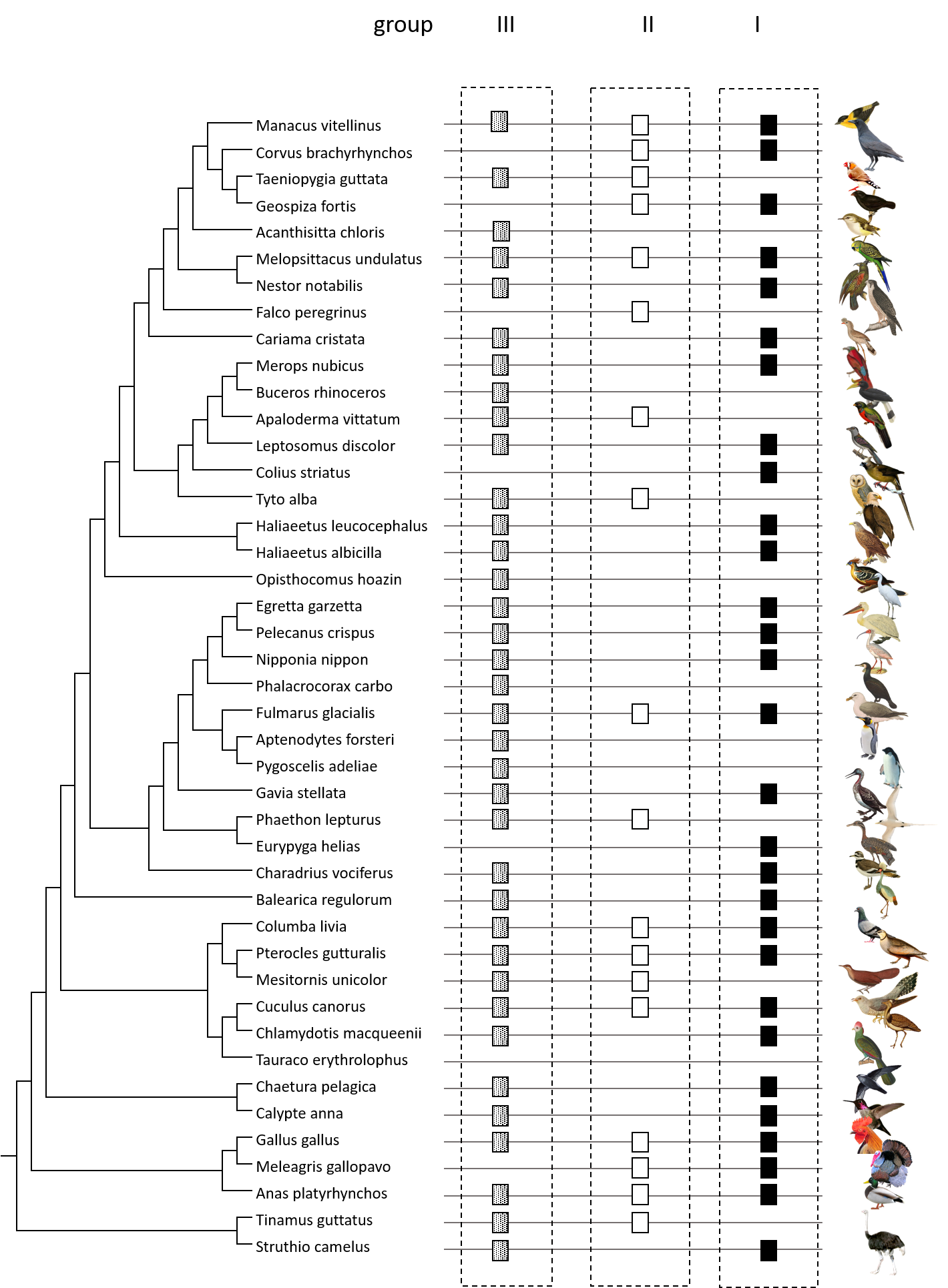

Supplement: S4 Fig — A phylogenetic tree depicting how UGT2 family genes are classified. (PNG) [file pone.0205266.s004.png]
